# *BRCA1*, *BRCA2*, and *TP53* germline and somatic variants and clinicopathological characteristics of Brazilian patients with epithelial ovarian cancer

**DOI:** 10.1002/cam4.6729

**Published:** 2024-02-02

**Authors:** Caroline Stahnke Richau, Nicole de Miranda Scherer, Bruna Palma Matta, Elvismary Molina de Armas, Fábio Carvalho de Barros Moreira, Anke Bergmann, Claudia Bessa Pereira Chaves, Mariana Boroni, Anna Claudia Evangelista dos Santos, Miguel Angelo Martins Moreira

**Affiliations:** ^1^ Tumoral Genetics and Virology Program Instituto Nacional de Câncer Rio de Janeiro Brazil; ^2^ Bioinformatics and Computational Biology Laboratory Instituto Nacional de Câncer Rio de Janeiro Brazil; ^3^ Division of Pathology Instituto Nacional de Câncer Rio de Janeiro Brazil; ^4^ Clinical Epidemiology Instituto Nacional de Câncer Rio de Janeiro Brazil; ^5^ Gynecologic Oncology Department Cancer Hospital II, Instituto Nacional de Câncer Rio de Janeiro Brazil; ^6^ Present address: Hospital BP ‐ A Beneficência Portuguesa de São Paulo São Paulo Brazil

**Keywords:** *BRCA1*, *BRCA2*, homologous recombination repair pathway, ovarian cancer, somatic mutation, *TP53*

## Abstract

**Background:**

Approximately 3/4 of ovarian cancers are diagnosed in advanced stages, with the high‐grade epithelial ovarian carcinoma (EOC) accounting for 90% of the cases. EOC present high genomic instability and somatic loss‐of‐function variants in genes associated with homologous recombination mutational repair pathway (HR), such as *BRCA1* and *BRCA2*, and in *TP53*. The identification of germline variants in HR genes in EOC is relevant for treatment of platinum resistant tumors and relapsed tumors with therapies based in synthetic lethality such as PARP inhibitors. Patients with somatic variants in HR genes may also benefit from these therapies. In this work was analyzed the frequency of somatic variants in *BRCA1*, *BRCA2*, and *TP53* in an EOC cohort of Brazilian patients, estimating the proportion of variants in tumoral tissue and their association with progression‐free survival and overall survival.

**Methods:**

The study was conducted with paired blood/tumor samples from 56 patients. Germline and tumoral sequences of *BRCA1*, *BRCA2*, and *TP53* were obtained by massive parallel sequencing. The HaplotypeCaller method was used for calling germline variants, and somatic variants were called with Mutect2.

**Results:**

A total of 26 germline variants were found, and seven patients presented germline pathogenic or likely pathogenic variants in *BRCA1* or *BRCA2*. The analysis of tumoral tissue identified 52 somatic variants in 41 patients, being 43 somatic variants affecting or likely affecting protein functionality. Survival analyses showed that tumor staging was associated with overall survival (OS), while the presence of somatic mutation in *TP53* was not associated with OS or progression‐free survival.

**Conclusion:**

Frequency of pathogenic or likely pathogenic germline variants in *BRCA1* and *BRCA2* (12.5%) was lower in comparison with other studies. *TP53* was the most altered gene in tumors, with 62.5% presenting likely non‐functional or non‐functional somatic variants, while eight 14.2% presented likely non‐functional or non‐functional somatic variants in *BRCA1* or *BRCA2*.

## 1 INTRODUCTION

Ovarian cancer (OC) is one of the leading causes of death among gynecological malignancies, with 314,000 new cases and 207,500 deaths per year.^1^ In Brazil, this type of cancer is the eighth most incident and 7310 new cases per year were estimated for the period 2023‐2025.^2^ Ninety percent of ovarian cancer are derived from epithelial cells (epithelium of ovarian surface or ovarian tube), the remaining 10% are derived from germ cells or from granulosa‐theca cells, being 3/4 of OC cases diagnosed in advanced stages and associated with worse outcome.^3^ The epithelial ovarian cancer (EOC) is classified in two major types, based on distinct invasiveness capacity and aggressiveness: low‐grade epithelial carcinoma (type I) and high‐grade serous epithelial carcinoma (type II or HGSOC).^4^, ^5^ Type II epithelial ovarian carcinomas account for 90% of cases and are classified into serous, mucinous, endometrioid, clear cell, transitional cell (Brenner tumors), mixed, and undifferentiated subtypes.^6^, ^7^ These tumors may present high genomic instability, frequently presenting somatic loss‐of‐function variants in *TP53* gene and in genes associated with homologous recombination (HR) mutational repair pathway, such as *BRCA1* and *BRCA2*.^8^, ^9^

The presence of germline loss‐of‐function variants in *BRCA1* or *BRCA2* confers a predisposition for breast cancer (absolute risk of 60%–85%) and ovarian cancer (absolute risk of 15%–40%).^10^ It was estimated that 20%–25% of EOC cases are associated with the presence of pathogenic germline variants in *BRCA1/2* ^11^, ^12^ or in other genes associated with tumor suppression and/or DNA damage response (*TP53*, *STK11*, *PTEN*, *ATM*, and *CHEK2*).^13^ On the contrary, the presence of somatic variants in *TP53* is frequently reported, with 91% of sporadic EOC presenting loss‐of‐function *TP53* variants.^14^, ^15^, ^16^ Some authors have associated the presence of somatic variants in *TP53* with patient's outcome; however, there are still contradictory and inconsistent findings in respect to this point.^17^, ^18^, ^19^, ^20^, ^21^, ^22^ Presence of *BRCA1*/2 germline loss‐of‐function variants in patients diagnosed with EOC was associated with an improved survival.^23^

Most patients with OC are submitted to surgical intervention followed by platinum‐based chemotherapy.^24^, ^25^ The identification of germline variants in HR genes in EOC patients came to be relevant for treatment of platinum resistant tumors and relapsed tumors, due to the development of therapies with poly (ADP ribose) polymerase inhibitors (PARPi), which are based on synthetic lethality.^26^, ^27^, ^28^ PARPi have been used in the treatment of patients with pathogenic germline variants in *BRCA1/2*.^29^, ^30^, ^31^, ^32^ The use of PARP inhibitors may be not limited to patients with pathogenic germline mutations in *BRCA1*/*2*, those with HR deficiency identified by the presence of specific patterns of mutations and chromosomal structural aberrations could also be benefited.^27^, ^33^ Additionally, investigation of somatic genetic variants can contribute to the understanding of deleterious events that result in tumor therapy resistance and clonal evolution of EOC tumors.^34^

The aim of this work was to analyze the presence of somatic variants in *TP53*, *BRCA1*, and *BRCA2* in EOC by massive parallel sequencing, estimating the proportion of these variants in tumor samples and their association with progression‐free survival (PFS) and overall survival (OS). Previous studies carried out in the Brazilian population have focused in describing germline variants in *BRCA1/2* and *TP53* in EOC patients.^13^, ^35^, ^36^, ^37^, ^38^, ^39^, ^40^, ^41^ The present study was carried out in a cohort of Brazilian patients, using an integrated analysis of germline and somatic (tumoral) variants. Our data contribute to a better characterization of these tumors, in view of the ongoing development of therapies targeting tumors with functional deficiency in HR genes.

## 2 MATERIALS AND METHODS

### 2.1 Study cohort

Tumor biopsies and blood samples used in this work were initially selected from samples of 108 patients collected by the National Tumors Bank (BNT) at the Brazilian National Cancer Institute (INCA – Brazil) between 2007 and 2017. All patients signed an informed consent before the collection of tumor and blood samples by the National Tumor Bank. Biopsies and blood samples were frozen in liquid nitrogen and stored at ‐80°C. This study was approved by Research Ethics Committee of the Brazilian National Cancer Institute (CAAE 78305417.3.0000.5274). Patients were diagnosed with epithelial ovarian carcinoma, and 94/108 paired blood/tumor samples were available. After a histopathologic revision, biopsies presenting <60% of malignant cells (*n* = 38) were excluded. In total, this study was carried out with samples from 56 patients with paired blood/tumor samples, confirmed diagnosis of epithelial ovarian carcinoma and tumor representativeness (TR) ≥ 60%.

Clinicopathological data about age at diagnosis, histological subtype, tumor staging at diagnosis carried out according to The International Federation of Gynecology and Obstetrics (FIGO), family/personal cancer history of patients, treatment, disease progression, and last follow‐up or death were obtained from medical records. The time of PFS was calculated as the period from the diagnosis to the disease progression or last follow‐up. The OS was calculated as the period from the diagnosis to the date of death or last follow‐up, as suggested by Tuna et al.^20^

### 2.2 DNA isolation

Genomic DNA was isolated from frozen tumor tissue and blood samples. DNA was purified from ~25 mg of tumor tissue using the QIAamp® DNA Mini Kit (Qiagen, USA), according to manufacturer's instructions. DNA from buffycoat or PBMC was isolated with QIAamp® DNA Mini Kit (Qiagen, USA) or ReliaPrep™ gDNA Tissue Miniprep System (Promega, USA), according to manufacturers' instructions. DNA was quantified by spectrophotometry with NanoDrop 2000 UV Spectrophotometer (Thermo Scientific, Canton, GA, USA). Genomic DNA integrity was evaluated through 0.8% agarose gel electrophoresis.

### 2.3 Exons amplification by polymerase chain reaction (PCR)

Exons and intronic flaking regions (at least 10 bp) from *BRCA1*, *BRCA2*, and *TP53* were amplified by multiplex PCR or long‐range PCR as described in Matta et al.,^42^ but with modifications (see Appendix S1). All PCR products were purified with the PureLink™ PCR Purification (Invitrogen™, Thermo‐Fisher Corporation). DNA concentration was normalized to 0.4 ng/μL for library preparation and massive parallel sequencing.

### 2.4 Massive parallel sequencings and sequence data analysis

DNA libraries were prepared using Nextera XT DNA Library (Illumina, San Diego, USA), according to manufacturer's instructions. DNA libraries were quantified with Qubit® 3.0 Fluorometer (Life Technologies). Libraries from the same sample were multiplexed using a 3:1 ratio of tumor: blood libraries, to increase the depth of coverage of tumor samples. Massive parallel sequencing was performed in a single run on the MiSeq platform (Illumina, San Diego, USA), with 150 × 150 paired end reads.

Raw sequencing data were converted from BCL format to FASTQ using BaseSpace platform (Illumina). Data were processed for read quality using the Prinseq software, and reads with Qscore < 30 were excluded from analysis. The high‐quality reads were mapped to the reference sequences of the GRCh38/hg38 UCSC version of the *BRCA1* genes (NM_007294.3); *BRCA2* (NM_000059.3) and *TP53* (NM_000546.5) using the Burrows–Wheeler Aligner (BWA).^43^

After pre‐processing the data, amplicon and base coverage was estimated for all target regions. Variant calling of single nucleotide substitutions (SNPs) and insertions/deletions (indels) was performed using a custom bioinformatics pipeline adapted for the genetic panel of *BRCA1*, *BRCA2*, and *TP53*.^44^ This process was divided into two independent steps: (1) calling of germline variants using blood samples and (2) calling of somatic variants using paired samples (tumor and blood). The germline variant calling was carried out by using HaplotypeCaller method, available on the GATK4 website.^45^ To verify variant quality and to eliminate artifacts or false positive variants, the following filters were applied: QualByDepth, FisherStrand, StrandOddsRatio, RMSMappingQuality, MappingQualityRankSumTest, and ReadPosRankSumTest, according to GATK suggested parameters. Somatic variants were called with Mutect2 simultaneously using germline and somatic sequence reads. For filtering, somatic variants were used the FilterMutectCalls, which allows the identification of low allele frequencies (<10%) and the removal of germline events, artifacts, and possible tumor contamination by normal tissue.^46^

Germline variants with read depth < 30× (DP < 30×) and with alternative allele frequency <0.2 (for SNPs) or <0.25 (for Indels) were removed from the analysis. For tumoral samples, somatic variants with DP < 50×, with a minimum count of the alternative allele <10× (minALTcount < 10×) and localized in intronic position beyond the canonical splicing sites (±1/±2), were excluded. All identified variants were annotated using VEP from Ensembl.^47^

The proportion of tumoral cells with a somatic mutation (Adjusted Variant Allele Frequency, or VAF‐adj) were calculated according to Lawson et al.,^21^ by using the proportion of tumor cells relative to normal cell in the biopsy, that is, tumor representativeness (TR). The VAF‐adj in somatic samples was estimated as the proportion (in percentage) of the alternative allele in the biopsy relative to the proportion of tumoral cells (TR): VAF‐adj. = VAF × 100%/TR.

### 2.5 Variant classification

The pathogenicity classification of *BRCA1*, *BRCA2*, and *TP53* germline variants followed the joint recommendations of the American College of Medical Genetics and Genomics (ACMG) and the Association for Molecular *Pathology* (*AMP*),^48^ the Clinical Genome Resource (ClinGen) updates of such recommendations, as well as CanVIG‐UK guidelines for cancer susceptibility genes (v2.16), and gene‐specific recommendations from CanVIG‐UK (*TP53*: v1.5; *BRCA1/BRCA2*: v1.17) and ClinGen (*TP53*: v1.2).^49^, ^50^ Details on each germline classification criterion are described in Matta et al.^42^ Germline variants were classified in five categories: (a) pathogenic, (b) likely pathogenic, (c) variant of uncertain significance (VUS), (d) likely benign, and (e) benign.

Somatic variants were classified according to the joint recommendations of ClinGen, Cancer Genomics Consortium (CGC), and Variant Interpretation for Cancer Consortium (VICC),^51^ being categorized into (a) oncogenic, (b) likely oncogenic, (c) VUS, (d) likely benign, and (e) benign. To support this classification, we used the databases Cancer Hotspots and Catalogue of Somatic Mutations in Cancer (COSMIC).^52^, ^53^

In silico predictors suited for functional variant effect were also employed to categorize the somatic variants predicted as deleterious, uncertain, or tolerated: REVEL for missense variants^54^; BayesDel for missense and nonsense variants^55^; mutfunc for inframe indel variants^56^; SpliceAI for splicing variants^57^; and AutoPVS1 for frameshift, nonsense, or splicing variants.^58^ Cutoffs or criteria for this categorization were based on the references above and in Pejaver et al., for REVEL and BayesDel, and Tayoun et al. for AutoPVS1.^59^, ^60^ Additionally, a functional categorization was performed, based on functional studies curated by *TP53* Database R20 version, CanVIG‐UK, or obtained by literature searches.^61^, ^62^ Variants were then categorized as non‐functional if presented a loss‐of‐function (LOF) effect in at least one functional study; likely non‐functional, if variant effect was nonsense, frameshift, or splicing but no functional study was found to corroborate the predicted deleterious effect; functional, if there was a curated functional study showing a wild‐type effect or if variant was classified as polymorphism (gnomAD allele frequency >1%); otherwise, variant functional categorization was deemed uncertain.

### 2.6 Characterization of loss of heterozygosity

Germline variants classified as likely pathogenic or pathogenic were analyzed for loss of heterozygosity (LOH) by visual inspection of the reads in the paired tumoral sample, using the Integrative Genomics Viewer (IGV) tool. The presence and frequency of the alternative germline allele in the given tumoral sample were computed, and the VAF‐adj was estimated. LOH was considered when the VAF‐adj of the likely pathogenic or pathogenic germline allele in the tumoral sample was ≥80%.

### 2.7 Survival analyses

Association between progression‐free survival (PFS) or overall survival (OS) with clinical‐pathological characteristics and with the presence of somatic mutations in *TP53* grouped by functional categories was evaluated. The clinical‐pathological characteristics were grouped by tumor histological subtypes (HGSOC vs. other subtypes), tumor staging (I–II vs. III–IV), and age at diagnosis (<50 vs. ≥50 years of age at diagnosis). In respect to the presence of somatic mutations in *TP53*, patients were grouped in those with variants categorized as non‐functional or likely non‐functional versus those with variants categorized as uncertain or functional effect plus patients without *TP53* somatic mutations. Kaplan–Meier survival analysis and log‐rank tests were used to evaluate PFS and OS in relation to each variable. Univariate Cox‐regression analyses were performed to calculate relative risk, that is, hazard ratio (HR), and 95% CI of each variable in relation to clinical outcome (PFS or OS). Variables with significant relative risk in the univariate analyses (*p* < 0.05) were submitted to multivariate Cox‐regression analysis. Kaplan–Meier and Cox‐regression estimates were performed using SPSS software version 23 (SPSS, Inc., Chicago, IL, USA).

## 3 RESULTS

### 3.1 Patient clinicopathological characteristics and NGS metrics

Table 1 describes the clinicopathological characteristics of the cohort. Patients included in this study were diagnosed with EOC, the majority (34/56, 60.7%) with High Grade Serous Ovarian Carcinoma (HGSOC). The median age at diagnosis was 57 years (range, 31–94), being 23/56 (41.1%) and 14/56 (25.0%) diagnosed in FIGO stages III and IV, respectively. Thirteen patients (23.2%) had family history consistent with Hereditary Breast and Ovary Cancer Syndrome (HBOC), characterized by the presence of breast/ovary cancers in first and/or second‐degree relatives. Forty‐eight patients (85.4%) underwent total abdominal hysterectomy (TAH), bilateral salpingo‐oophorectomy (BSO), and omentectomy cytoreduction surgeries. Fifteen (26.8%) patients received neoadjuvant chemotherapy with carboplatin and paclitaxel. Most patients (46/56, 82.1%) were submitted to adjuvant chemotherapy regimen based on carboplatin and paclitaxel, and 19 patients (33.9%) with disease progression received additional regimens of adjuvant chemotherapy with gemcitabine, topotecan, or paclitaxel.

**TABLE 1:** Characterization of clinicopathological characteristics of the 56 patients included in the study.

	No (range)	%
Patient characteristics		
Number of patients included	56	100
Age at diagnosis, median (range)	57 (31–94)	
Histological subtype		
High‐grade serous ovarian carcinoma	34	60
Low‐grade serous ovarian carcinoma	2	04
Mucinous carcinoma	8	14
Clear cell carcinoma	7	13
Endometrioid carcinoma	3	05
Mixed tumor^a^	1	02
Mixed tumor^b^	1	02
Staging		
I	16	29
II	3	05
III	23	41
IV	14	25
HBOC family history		
Yes	13	23
No	43	77
Personal history of other cancers^c^		
Yes	4	07
No	52	93
Cytoreduction surgery		
Primary Debulking Surgery (TAH + BSO + Omentectomy)	36	64
Primary Debulking Surgery (Other Cytoreduction procedures)	3	6
Interval Debulking Surgery (TAH + BSO + Omentectomy)	12	21
Interval Debulking Surgery (Other Cytoreduction procedures)	5	9
Neoadjuvant chemotherapy		
Yes	15	27
No	41	73
Adjuvant chemotherapy		
Carboplatin and Paclitaxel	29	52
Carboplatin and Paclitaxel + Additional regimens^d^	17	30
Gemcitabine	2	4
None adjuvant chemotherapy	8	14
Disease progression		
Yes	36	64
No	20	36
Deaths		
Yes	36	64
No	20	36
OS in months, median (range)	52 (1–131)	
PFS in months, median (range)	22 (1–131)	

Abbreviations: BSO, bilateral salpingo‐oophorectomy; HBOC, Hereditary Breast and Ovarian Cancer Syndrome; OS, Overall Survival; PFS, progression‐free survival; TAH, total abdominal hysterectomy.

^a^ Tumor with histological subtypes HGSOC and Mucinous carcinoma.

^b^ Tumor with mixed histological subtypes HGSOC and Clear cell carcinoma.

^c^ Cases who had others type of cancer (colorectal cancer, melanoma and basal cell carcinoma) before ovarian cancer diagnosis.

^d^ Additional regimens of adjuvant chemotherapy with gemcitabine, topotecan, or paclitaxel.

Genomic DNA was isolated from blood and biopsies and submitted to massive parallel sequencing of *BRCA1*, *BRCA2*, and *TP53* genes, resulting in average depth coverage for tumor samples of 2,330,744 reads for *BRCA1* (range: 442,640–7,475,773), 1,965,145 reads for *BRCA2* (range: 227,065–5,377,818), and 1,505,640 reads for *TP53* (range: 961,945–2,375,777). In blood samples, average depth coverage was 335,995 reads for *BRCA1* (range: 59,167–2,166,925), 287,289 reads for *BRCA2* (range: 71,233–818,111), and 381,899 reads for *TP53* (range: 308,361–489,603).

### 3.2 Germline variants

A total of 26 distinct germline variants were found, all located in coding exons of *BRCA1*, *BRCA2*, and *TP53* (Appendix S2). Missense substitutions were the most frequent, corresponding to 22 variants (five in *BRCA1*, 14 in *BRCA2*, and three in *TP53*) followed by two nonsense substitutions (both in *BRCA1*) and two frameshift variants (both in *BRCA2*).

In respect to variant pathogenicity classification, 18 variants were classified as benign (three in *BRCA1*, 12 in *BRCA2*, three in *TP53*), two as VUS (both in *BRCA2*), and six as likely pathogenic or pathogenic (four in *BRCA1* and two in *BRCA2*). The VUS *BRCA2*:c.6443C>A has conflicting interpretation of pathogenicity in ClinVar (either likely benign/benign or VUS), and the VUS *BRCA2*:c.6281A>G was predicted to be benign by two in silico predictors (REVEL and BayesDel; see Appendix S2).

Among the variants classified as pathogenic/likely pathogenic, the nonsense substitution *BRCA1* c.1387A>T (p.Lys463*) was not reported before in dbSNP or ClinVar. This nonsense variant was interpreted as likely pathogenic, following the PVS1 and PMS supporting criteria of the ACMG recommendations^48^ ^,49^: null variant (nonsense, frameshift, canonical ±1 or 2 splice sites, initiation codon, single or multi‐exon deletion) in a gene where loss of function (LOF) is a known mechanism of disease, and absent from controls (absent in gnomAD database). This variant was in exon 11, in the Serine‐rich Domain Associated with the BRCT and in the Ovarian Cancer Cluster Region (c.670‐ c.4096), a region where presence of pathogenic variants increases the risk for developing ovarian cancer.^63^ The patient carrying this variant was diagnosed with HGSOC at 62 years old and did not report familial history of cancers associated with HBOC in first‐ and second‐degree relatives.

The VUS and pathogenic/likely pathogenic germline variants are listed in Table 2, and their respective positions in respect to BRCA1 and BRCA2 protein domains are shown in Figure S1.

**TABLE 2:** Germline variants in *BRCA1* and *BRCA2* genes classified as pathogenic, likely pathogenic, or VUS, and evidence for LOH in tumoral tissue.

Patient	Exon	dbSNP	HGVSc	HGVSp	ClinVar	ACMG/AMP classification	VAF blood/VA*F*_adj_ tumor
*BRCA1*							
1	11	‐	c.1387A>T^a^	p.Lys463*	‐	LP	42%/89%^b^
66	11	rs80357136	c.3403C>T	p.Gln1135*	P	P	52%/86%^b^
12	14	rs80357389	c.4484G>T	p.Arg1495Met	P	P	43%/84%^b^
67	16	rs80357390	c.4964C>T	p.Ser1655Phe	P/LP	LP	51%/100%^b^
42	16	rs80357390	c.4964C>T	p.Ser1655Phe	P/LP	LP	53%/34%
*BRCA2*							
62	10	rs80359264	c.1138del	p.Ser380ValfsTer19	P	P	48%/83%^b^
64	11	rs80359535	c.5771_5774del	p.Ile1924ArgfsTer38	P	P	48%/100%^b^
62	11	rs397507838	c.6281A>G	p.Tyr2094Cys	VUS	VUS	58%/38%
67	11	rs80358880	c.6443C>A	p.Ser2148Tyr	VUS/LB	VUS	50%/56%

*Note*: ACMG/AMP classification considers ClinGen updates of ACMG/AMP guidelines, as well as CanVIG‐UK guidelines for cancer susceptibility genes (v2.17) (see Section 2).

Abbreviations: LB, likely benign; LP, likely pathogenic; P, pathogenic; VUS, variant of uncertain significance.

^a^ A variant not reported in dbSNP.

^b^ Patients with tumors presenting LOH (Loss of Heterozygosity or loss of the reference allele), what means tumors with VAF‐adjusted (VAF_adj_) > 80%.

### 3.3 Somatic variants

**FIGURE 1:**
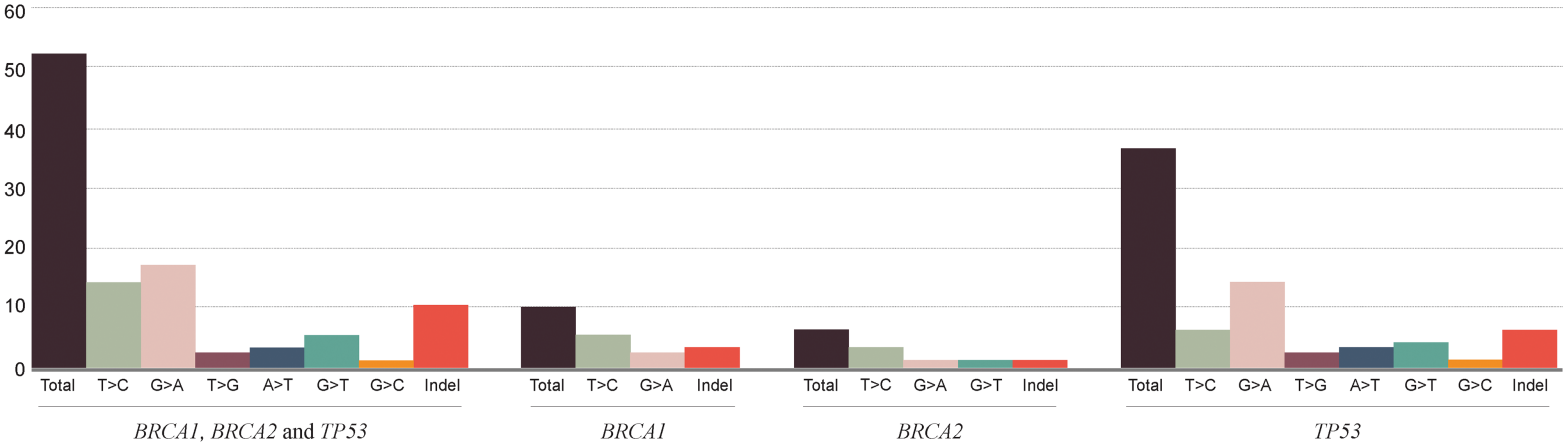
Number of nucleotide substitution by different categories (T>C or A>G; G>A or C>T; T>G or A>C; A>T or T>A; G>T or C>A; G>C or C>G) and indels for *BRCA1*, *BRCA2*, and *TP53* in tumoral samples. The first cluster shows the nucleotide substitution for the three genes and the others show for each gene separately.

A total of 52 somatic variants were found in 41/56 patients (Appendix S2). Forty‐two variants were single base substitutions (SBSs) being seven in *BRCA1*, five in *BRCA2* and 30 in *TP53*. Ten variants were small indels, being three in *BRCA1*, one in *BRCA2*, and six in *TP53*. The G>A or C>T substitution was the most frequent (*n* = 17 substitutions) followed by T>C or A>G with 14 substitutions (Figure 1). Somatic variant classification resulted in 41 classified as likely oncogenic/oncogenic, four as VUS, and seven as likely benign/benign. In respect to the possible functionality of the somatic variants, seven variants were categorized as functional (Appendix S2), 31 as non‐functional, 10 as likely non‐functional, and four were categorized as uncertain functional effect (Table 3).

**TABLE 3:** Tumor representativeness and functional classification of somatic variants in *BRCA1*, *BRCA2*, and *TP53* genes classified as oncogenic, likely oncogenic, or VUS.

Patient	Exon [Intron]	dbSNP	HGVSc	HGVSp	TR (%)/VAF_adj_.(%)	ClinGen/CCG/VICC Classification	Hotspot	Functional prediction	Functional categorization
*BRCA1*									
4(2)	10	rs80357537	c.668del	p.Lys223ArgfsTer11	100/2	Likely Oncogenic	No	Deleterious	Likely NF
10(2)	11	–	c.1952A>G	p.Lys651Arg	100/0.5	VUS	No	Uncertain	Uncertain
15(1)	11	rs886040056	c.2637del	p.Glu880ArgfsTer13	100/5	Likely Oncogenic	No	Deleterious	Likely NF
67(3)^a^	11	rs886040056	c.2637del	p.Glu880ArgfsTer13	80/4	Likely Oncogenic	No	Deleterious	Likely NF
8(1)	11	–	c.3600_3619del	p.Gly1201GlufsTer11	70/12	Likely Oncogenic	No	Deleterious	Likely NF
57(2)	20	rs80356937	c.5212G>A	p.Gly1738Arg	90/89	Likely Oncogenic	No	Deleterious	Non‐functional
*BRCA2*									
35(2)	10	rs80358438	c.1528G>T	p. Glu510*	60/100	Likely Oncogenic	No	Deleterious	Likely NF
34(1)	11	rs80359406	c.3860del	p.Asn1287IlefsTer6	70/29	Likely Oncogenic	No	Deleterious	Likely NF
12(2)^a^	11	rs1555284238	c.5462A>G	p.Lys1821Arg	100/2	VUS	No	Tolerated	Uncertain
33(2)	18	rs757206472	c.8008T>C	p.Ser2670Pro	100/1	VUS	No	Deleterious	Uncertain
66(1)^a^	21	rs397508002	c.8680C>T	p.Gln2894*	100/9	Likely Oncogenic	No	Deleterious	Likely NF
*TP53*									
17(1)	4	rs587783062	c.267del	p.Ser90ProfsTer33	100/90	Likely Oncogenic	No	Deleterious	Likely NF
64(1)^a^	4	rs2073465664	c.272G>A	p.Trp91*	70/47	Oncogenic	Yes	Deleterious	Non‐functional
49(1)	4	rs1555526478	c.372C>A	p.Cys124*	90/100	Likely Oncogenic	No	Deleterious	Non‐functional
52(1)	5	rs730881999	c.380C>T	p.Ser127Phe	100/47	Likely Oncogenic	Yes (ov)	Deleterious	Non‐functional
4(2)	5	rs1057519978	c.421T>G	p.Cys141Gly	100/60	Likely Oncogenic	Yes	Deleterious	Non‐functional
5(1)	5	rs730882019	c.455del	p.Pro152ArgfsTer18	100/64	Likely Oncogenic	Yes	Deleterious	Non‐functional
50(1)	5	rs121912654	c.469G>T	p.Val157Phe	60/75	Oncogenic	Yes (ov)	Deleterious	Non‐functional
60(1)	5	rs148924904	c.488A>G	p.Tyr163Cys	80/88	Oncogenic	Yes (ov)	Deleterious	Non‐functional
9(1)	5	rs730882001	c.493C>T	p.Gln165*	100/90	Oncogenic	Yes	Deleterious	Non‐functional
55(1)	5	rs730882001	c.493C>T	p.Gln165*	80/59	Oncogenic	Yes	Deleterious	Non‐functional
33(2)	5	rs876660754	c.517G>T	p.Val173Leu	100/56	Oncogenic	Yes (ov)	Deleterious	Non‐functional
65(1)	5	rs28934578	c.524G>A	p.Arg175His	80/71	Oncogenic	Yes (ov)	Deleterious	Non‐functional
1(1)^a^	5	rs1057519991	c.536A>G	p.His179Arg	100/69	Oncogenic	Yes (ov)	Deleterious	Non‐functional
32(1)	[5]	–	c.559+2T>G	p.?	100/83	Likely Oncogenic	No	Deleterious	Likely NF
12(2)^a^	6	rs786201838	c.578A>G	p.His193Arg	100/61	Oncogenic	Yes (ov)	Deleterious	Non‐functional
3(1)	6	rs760043106	c.584T>C	p.Ile195Thr	100/73	Oncogenic	Yes (ov)	Deleterious	Non‐functional
27(1)	6	rs397516436	c.637C>T	p.Arg213*	90/100	Oncogenic	Yes (ov)	Deleterious	Non‐functional
7(1)	6	rs121912666	c.659A>G	p.Tyr220Cys	100/59	Oncogenic	Yes (ov)	Deleterious	Non‐functional
67(3)^a^	7	rs765848205	c.710T>A	p.Met237Lys	80/39	Oncogenic	Yes (ov)	Deleterious	Non‐functional
54(1)	7	rs587782664	c.711G>C	p.Met237Ile	100/64	Oncogenic	Yes (ov)	Deleterious	Non‐functional
35(2)	7	–	c.732_733insCATGCG	p.Gly244_Gly245insHisAla	60/90	VUS	Yes	Deleterious	Uncertain
57(2)	7	rs28934575	c.733G>A	p.Gly245Ser	90/71	Oncogenic	Yes (ov)	Deleterious	Non‐functional
48(1)	7	rs121912651	c.742C>T	p.Arg248Trp	100/67	Oncogenic	Yes (ov)	Deleterious	Non‐functional
10(2)	7	rs11540652	c.743G>A	p.Arg248Gln	100/74	Oncogenic	Yes (ov)	Deleterious	Non‐functional
11(1)	7	rs730882027	c.752T>A	p.Ile251Asn	100/78	Likely Oncogenic	Yes	Deleterious	Non‐functional
31(1)	7	rs1064794309	c.764_766del	p.Ile255del	80/51	Likely Oncogenic	Yes	Deleterious	Non‐functional
30(1)	7	rs1427471466	c.780del	p.Ser261ValfsTer84	100/75	Likely Oncogenic	No	Deleterious	Non‐functional
63(1)	[7]	rs1555525367	c.783‐1G>T	p.?	100/82	Likely Oncogenic	No	Deleterious	Likely NF
56(1)	8	rs193920774	c.797G>A	p.Gly266Glu	90/100	Oncogenic	Yes	Deleterious	Non‐functional
16(1)	8	rs28934576	c.818G>A	p.Arg273His	100/95	Oncogenic	Yes (ov)	Deleterious	Non‐functional
67(3)^a^	8	rs876659802	c.833C>T	p.Pro278Leu	80/44	Oncogenic	Yes (ov)	Deleterious	Non‐functional
6(1)	8	rs587781525	c.842A>T	p.Asp281Val	100/36	Oncogenic	Yes (ov)	Deleterious	Non‐functional
43(1)	8	rs28934574	c.844C>T	p.Arg282Trp	100/3	Oncogenic	Yes (ov)	Deleterious	Non‐functional
23(1)	8	rs786201059	c.856G>A	p.Glu286Lys	80/15	Oncogenic	Yes (ov)	Deleterious	Non‐functional
2(1)	8	rs121913344	c.916C>T	p.Arg306*	100/50	Oncogenic	Yes (ov)	Deleterious	Non‐functional
42(1)^a^	9	rs2073149039	c.988del	p.Leu330PhefsTer15	100/16	Likely Oncogenic	No	Deleterious	Likely NF

Abbreviations: Likely NF, Likely non‐functional; TR (%), tumor representativeness; VUS, variant of uncertain significance.

^a^ Denotes patients with germline pathogenic or likely pathogenic variant (see Appendix S2). VAF_adj._(%) means variant allele frequency adjusted for tumor representativeness (VAF_adj._ = VAF × 100%/ RT). ClinGen/CCG/VICC classification follows the guidelines for classification of pathogenicity of somatic variants in cancer (oncogenicity). Cancer hotspots information was obtained from cancerhotspots.org, and (ov) indicates that the somatic variant was previously reported in ovary/fallopian tube cancers. Functional prediction was based on prediction programs suited for each variant effect: REVEL (missense); SpliceAI (splicing); BayesDel (nonsense); AutoPVS1 (frameshift, nonsense or splicing); mutfunc db (inframe indel). Functional categorization was mainly based on functional studies that tested the variant effect on protein function (for details see Methods); studies were curated by CanVIG‐UK guidelines, TP53 Database, and literature searches.

For *BRCA1* and *BRCA2*, 16 somatic variants were identified in 14 patients being six variants (in three patients) categorized as functional (Appendix S2), one variant (in one patient) as non‐functional, six variants (in seven patients) as likely non‐functional, and three variants (in three patients) as uncertain functional effect (Table 3). Among these variants, two, in *BRCA1*, were not reported in ClinVar or dbSNP: c.3600_3619del(p.Gly1201‐GlufsTer11) and c.1952A>G(p.Lys651.Arg). The first variant, a frameshift deletion in exon 11 results in an early stop codon being predicted to be a likely oncogenic variant. The missense variant c.1952A>G(p.Lys651.Arg) was classified as a VUS as there were no supporting data based on functional studies, in silico predictions, or cancer databases. The allelic frequency of somatic variants adjusted by the frequency of tumoral cells in the biopsies (VAF‐adj) ranged from 0.5% to 89% for *BRCA1* (median 4.5%) and 1%–100% for *BRCA2* (median 9%). The position of each somatic variant in respect to exons and protein domains is shown in Figure S2.

A total of 36 *TP53* somatic variants were found in tumors of 35 patients (Table 3), with 34 variants (in 34 patients) categorized as non‐functional or likely non‐functional, one variant as functional (c.85A>G (p.Asn29Asp)), and one as of uncertain functional effect (c.732_733insCATGCG (p.Gly244_Gly245insHisAla)). This inframe insertion of six nucleotides occurred between codons considered mutational hotspots (Gly244 and Gly245) and was not previously reported in dbSNP (see Table 3 and Appendix S2). The variant c.559+2T>G was also not reported in dbSNP and was considered likely oncogenic and non‐functional since it affects a canonical splicing site and presents a deleterious in silico prediction (Appendix S2). Both non‐reported variants were found in patients diagnosed at 60 years old (c.732_733insCATGCG (p.Gly244_Gly245insHisAla)) and 64 years old (c.559+2T>G), both with HGSOC stage III, and without familial history of cancer.

Indicative of loss of heterozygosity (LOH) was found in tumors from six patients carrying *BRCA1* or *BRCA2* germline variants (Table 2). For *BRCA1* carriers, LOH was observed for the following variants: c.1387A>T (p.Lys463*) classified as likely pathogenic, c.3403C>T (p.Gln1135*) and c.4484G>T (p.Arg1495Met) classified as pathogenic, and for one carrier of c.4964C>T (p.Ser1655Phe), a variant classified as likely pathogenic. For *BRCA2*, LOH was observed in tumors of two patients carrying the pathogenic variants c.1138del (p.Ser380ValfsTer19) and c.5771_5774del (p.Ile1924ArgfsTer38). On the contrary, no evidence of LOH was found for carriers of the germline variants *BRCA2*:c.6281A>G (p.Tyr2094Cys) and *BRCA2*:c.6443C>A (p.Ser2148Tyr) classified as VUS, and for one carrier of the likely pathogenic variant *BRCA1*:c.4964C>T (p.Ser1655Phe). Interestingly, the carrier of VUS *BRCA2*:c.6281A>G (p.Tyr2094Cys) is also a carrier of the pathogenic variant *BRCA2*: c.1138del with LOH.

### 3.4 Survival analyses

The univariate analysis found significant associations of progression‐free survival (PFS) with histological subtype and tumor staging, where HGSOC and stages III–IV were associated with worse progression (Table 4; Kaplan–Meier curves in Figure S3). However, after the adjusted Cox‐regression, only tumor staging maintained a significant association (*p* = 0.002; HR = 5.218, 95% CI = 1.867–14.587). Considering overall survival (OS), only tumor staging presented a significant association (*p* = 0.001; HR = 4.948; 95% CI = 1.906–12.845), where stages III‐IV were associated with worse overall survival (Table 4; Kaplan–Meier curves in Figure S3). There were no significant associations of PFS or OS with the presence of tumor variants categorized as non‐functional/likely non‐functional in *TP53*.

**TABLE 4:** Progression‐free survival and overall survival analysis in respect to clinicopathologic characteristics and presence of somatic variants in *TP53*.

	PFS (mean)	PFS *p*‐value^a^	PFS HR (*p*‐value)	PFS 95% CI	OS (mean)	OS *p*‐value^a^	OS HR (*p*‐value)	OS 95% CI
Univariate analysis								
Histological subtype								
Other (*N* = 22)	78.5 (±11.5)	0.018	2.4 (0.022)	1.1–5.3	70.6 (±11.1)	0.343	1.4 (0.347)	0.7–2.9
HGSOC (*N* = 34)	40.8 (±7.8)				61.7 (±7.4)			
Tumor staging								
I–II (*N* = 19)	97.1 (±9.1)	<0.001	5.4 (<0.001)	2.2‐13.3	97.6 (±9.9)	<0.001	4.9 (0.001)	1.9‐12.8
III–IV (*N* = 37)	34.9 (±7.3)				49.4 (±6.3)			
Age at diagnosis								
<50 years (*N* = 15)	58.9 (±13.3)	0.781	1.0 (0.673)	0.9–1.0	74.9 (±13.0)	0.403	1.0 (0.207)	1.0–1.1
≥50 years (*N* = 41)	51.7 (±8.0)				60.2 (±6.9)			
*TP53* somatic variant functional classification^b^								
Functional (*N* = 22)	63.1 (±11.7)	0.285	1.5 (0.291)	0.7–3.0	59.0 (±9.4)	0.664	0.9 (0.665)	0.4–1.7
Non‐ functional (*N* = 34)	49.8 (±8.6)				68.3 (±8.3)			
PFS multivariate analysis of histological subtype and tumor staging								
Histological subtype	**–**	**–**	1.1 (0.896)	0.4–2.5	**–**	**–**	**–**	**–**
Tumor staging	**–**	**–**	5.2; (0.002)	1.9–14.6	**–**	**–**	**–**	**–**

*Note*: HR and 95% CI refers to hazard ratio and confidence interval from Univariate Cox‐regression analyses.

Abbreviations: OS, overall survival; PFS, progression‐free survival.

^a^ *p*‐Value from Log‐rank test.

^b^ The presence of somatic mutations in *TP53*, patients were grouped in those with variants classified as non‐functional or likely non‐functional variants versus patients those with variants classified as uncertain effect/functional variants and patients without somatic variants in *TP53*.

## 4 DISCUSSION

In this study, germline pathogenic or likely pathogenic variants were detected in *BRCA1* and *BRCA2* and were found in 12.5% (7/56) of patients. This frequency is lower than those reported in studies carried out in other countries, with patients with *BRCA1* and *BRCA2* germline variants and not selected for hereditary syndromes: 14.6%–17.5% in United States^64^, ^65^; 21.5% in Black Americans^66^; and 22% in Chinese population.^67^ Three other reports analyzing Brazilian patients also found a higher frequency of germline pathogenic variants in *BRCA1* and *BRCA2*: 19%,^35^ 20%,^68^ and 27.2%.^69^ Interestingly, in our set of patients, no patient was found with one of the pathogenic germline variants frequently reported in the Brazilian population: *BRCA1*:c.5266dupC.^35^, ^38^, ^68^, ^70^ Another recurrent pathogenic germline variant not found in our cohort is *TP53*:c.1010G>A (p.R337H), which is considered a Brazilian founder mutation, with a populational frequency of ~0.3% in Brazilian Southern region.^71^, ^72^ While this *TP53* variant is associated with hereditary breast and ovarian syndrome, it has been reported mainly in breast cancer cases.^42^, ^73^

In this work, the analysis of tumor sequences showed loss of the germline reference allele (LOH) in all patients carrying germline variants classified as pathogenic or likely pathogenic, except for one carrier of the variant *BRCA1*:c.4964C>T (p.Ser1655Phe). This variant was found in two patients: LOH was detected in one (VAF = 80%; and VAF‐adj = 100% in the tumor biopsy), but no evidence of LOH was found in the other (VAF = VAF‐adj = 34% in the tumor biopsy). This variant is located in the BRCT‐1 domain, and functional assays showed that p.Ser1655Phe impairs the interaction of *BRCA1* with ABRAXAS, BRIP1, and CtIP proteins, acting in the HR pathway.^74^ However, this variant was considered as moderately deleterious in functional assays affecting the transduction of DNA damage signals.^75^ Interestingly, the patient carrying the germline variant *BRCA1* c.1387A>T (p.Lys463*), at the *BRCA1* Ovarian Cancer Cluster Region, classified as likely pathogenic (accordingly to ACMG) and presenting LOH in the tumoral tissue, did not present familial or personal cancer history consistent with HBOC.

Considering somatic variants, the G>A or C>T substitution was the most recurrent mutation (17/42), frequently occurring in cytosines present in CpG sites (8/17), resulting in missense or nonsense substitutions. These substitutions are associated with the deamination of 5‐methylcytosine in positions considered methylation hotspots.^76^, ^77^ This substitution pattern characterizes the 1A/B mutational signature, as defined by,^78^, ^79^ and is frequently reported in other cancer types, being positively correlated with age.

For *BRCA1* and *BRCA2*, somatic variants classified as oncogenic or likely oncogenic were detected in low frequency: only four in *BRCA1* (in five patients) and three in *BRCA2* (in three patients). All were nonsense or frameshift variants, except for one in *BRCA1* gene: the missense somatic variant c.5212G>A (p.Gly1738Arg). This variant was categorized as non‐functional, and the carrier presented a somatic VAF‐adjusted of 89%. Functional assays characterized a deleterious effect of this alteration, due to its location in the C‐terminal region of *BRCA1*, between the BRCT‐1 and BRCT‐2 domains, which deregulates the functional activity of the protein.^80^, ^81^

The prevalence of *TP53* somatic variants categorized as non‐functional or likely non‐functional, which may disturb p53 function, was 62.5% (35/56) of all patients, and 64.7% (22/34) of HGSOC patients. The frequency of *TP53* somatic variants in the present study was lower than the 68%–96% frequency found in other studies.^82^, ^83^, ^84^, ^85^ Most *TP53* somatic variants found here were missense substitutions spanning exons 5 to 8, which correspond to the DNA‐binding domain (amino acid residues 95–288).^86^, ^87^ Nineteen of these missense somatic variants are frequently reported in ovarian tumors, occurring at codons considered cancer hotspots,^88^, ^89^ and presented VAF‐adjusted ranging from 3% to 100%. All these missense variants were found to reduce/inactivate the transcriptional activity of TP53 protein^90^, ^91^ and/or to decrease/abolish the p53 antiproliferative activity, as measured by cell growth.^92^, ^93^ Interestingly, of the five *TP53* nonsense somatic variants found in this study, four are also described as cancer hotspots^89^ and were caused by the single G>A or C>T substitutions that characterize the 1A/B mutational signature.^78^, ^79^

In this work, most of the somatic variants (28/36) categorized as non‐functional or likely non‐functional presented a VAF‐adjusted ≥50%, indicating a prevalence of tumoral cells with non‐functional *TP53*. In the biallelic context, when VAF‐adjusted is ~50%, the presence of a non‐functional TP53 protein can affect the function of the wild‐type *TP53*, contributing to tumor progression (e.g., variants present in the oligomerization domain).^94^, ^95^, ^96^ As none of the cases presented a germline variant classified as likely oncogenic or oncogenic in *TP53*, it can be presumed that somatic alterations in *TP53* could be drivers of tumorigenesis. This hypothesis is also corroborated by the high frequency of *TP53* somatic variants in premalignant lesions of the epithelium of the ovarian or fallopian tube.^97^ However, studies about driver and passenger variants in *TP53* remain a challenge, because alterations in this gene are subjected to multiple selective pressures during tumor evolution.^98^

Considering the survival analyses, tumor staging III/IV were associated with worse overall survival, while late tumor staging and HGSOC were associated with a worse progression‐free survival, which is consistent with previous studies.^84^, ^99^ On the contrary, the presence in *TP53* of somatic non‐functional/likely NF variants was not associated with overall and progression‐free survival, agreeing with Ahmed et al., Tuna et al., and Ghezelayagh et al.^18^, ^20^, ^22^ Nevertheless, in a larger cohort (791 HGSOC samples from TCGA and other sample sets), Tuna et al.^20^ found that a subgroup of tumors with three variants in mutational hotspot sites (Y163C/G266/R282) was associated with worse OS in comparison to other *TP53* mutations. In our work, as in other similar studies, the small sample size might have obscured the association between non‐functional/likely ‐NF *TP53* somatic variants and patient outcome. Therefore, *TP53* mutational status could not be independently analyzed from tumor characteristics, like histological subtype and tumor staging, which are known to be associated with patient survival.

The identification of non‐functional or likely non‐functional somatic variants in *BRCA1/2*, in patients without germline variants in these genes, could aid in the detection of patients who may benefit from PARP inhibitors (PARPi) targeted therapies.^26^, ^27^, ^29^ For cases with somatic mutations, there are clinical studies that analyze the treatment response and patient survival after the administration of PARPi.^100^ Some investigations report the clinical benefit of carriers of somatic mutations with the use of PARPi and that the responses to treatment are similar between cases with germline or somatic alterations.^101^, ^102^, ^103^ According to these results, the patients with somatic mutations in *BRCA1/2*, detected in this study, could benefit from PARPi targeted therapy, since most cases are HGSOC present 100% representativeness in the tumor and, therefore, low levels of contamination by normal cells.

## 5 CONCLUSION

In the present study, the parallel sequencing analyses of normal and tumoral tissue allowed the identification of variants exclusively present in tumoral samples. *TP53* was the most altered gene, as expected, with 35 patients (62.5%) presenting likely oncogenic or oncogenic variants, while eight patients (14.2%) presented likely oncogenic variants in *BRCA1* and *BRCA2* genes. The simultaneous analysis of tumor and germline samples allowed the identification of somatic variants present in low frequency (<10%), and the detection of LOH in tumors from six patients with germline pathogenic/likely pathogenic variants in *BRCA1* or *BRCA2*. In addition, the frequency (12.5%) of germline pathogenic or likely pathogenic variants in *BRCA1*/*BRCA2* was lower in comparison with other works. In respect to somatic variants, our analyses did not show any association between presence of oncogenic/loss‐of‐function variants in *TP53* and OS or PFS. To our knowledge, this work was the first to carry out an integrated analysis of germline and somatic variants for the *BRCA1*, *BRCA2*, and *TP53* genes in a Brazilian cohort of epithelial ovarian cancer patients, and to evaluate possible associations between tumor mutational profile and survival outcomes in the Brazilian population.

## AUTHOR CONTRIBUTIONS

**Caroline Stahnke Richau:** Data curation (equal); formal analysis (equal); methodology (equal); validation (equal); visualization (equal); writing – original draft (equal); writing – review and editing (equal). **Nicole de Miranda Scherer:** Data curation (equal); methodology (equal); software (equal); validation (equal); visualization (equal); writing – review and editing (equal). **Bruna Palma Matta:** Conceptualization (equal); data curation (equal); methodology (equal); validation (equal); writing – review and editing (equal). **Elvismary Molina de Armas:** Data curation (equal); methodology (equal); validation (equal); visualization (equal); writing – review and editing (equal). **Fábio Carvalho de Barros Moreira:** Data curation (equal); methodology (equal); writing – review and editing (equal). **Anke Bergmann:** Methodology (equal); writing – review and editing (equal). **Claudia Bessa Pereira Chaves:** Conceptualization (equal); writing – review and editing (equal). **Mariana Boroni:** Data curation (equal); methodology (equal); writing – review and editing (equal). **Anna Claudia Evangelista dos Santos:** Conceptualization (equal); data curation (equal); formal analysis (equal); project administration (equal); writing – review and editing (equal). **Miguel Angelo Martins Moreira:** Conceptualization (equal); formal analysis (equal); funding acquisition (equal); project administration (equal); supervision (equal); writing – original draft (equal); writing – review and editing (equal).

## FUNDING INFORMATION

This work was supported by Brazilian Research Council (CNPQ, grant: 304339/2018‐0), Carlos Chagas Filho Research Support Foundation of the State of Rio de Janeiro (FAPERJ, grants: 200.928/2021 and E‐26/211.309/2021), and Ministry of Health (Brazil), Brazilian National Cancer Institute (INCA‐Brazil, intramural grants), and Swiss Bridge Foundation.

## CONFLICT OF INTEREST STATEMENT

The authors declare no conflict of interest.

## ETHICS STATEMENT

This work was approved by the institutional Research Ethics Committee (CAAE 78305417.3.0000.5274).

## Supporting information


Figure S1

Figure S2

Figure S3

Appendix S1

Appendix S2

Appendix S3

## Data Availability

Massive parallel sequencing data (BAM files) are available at DDBJ (DNA Data Bank of Japan) under the accession number PRJDB16141.
