# Supplementary figures and images for: BRCA1, BRCA2, and TP53 germline and somatic variants and clinicopathological characteristics of Brazilian patients with epithelial ovarian cancer
Source: Cancer Med. 2024 Feb 2;13(3):e6729. doi: 10.1002/cam4.6729 (PMC10905552; doi:10.1002/cam4.6729)

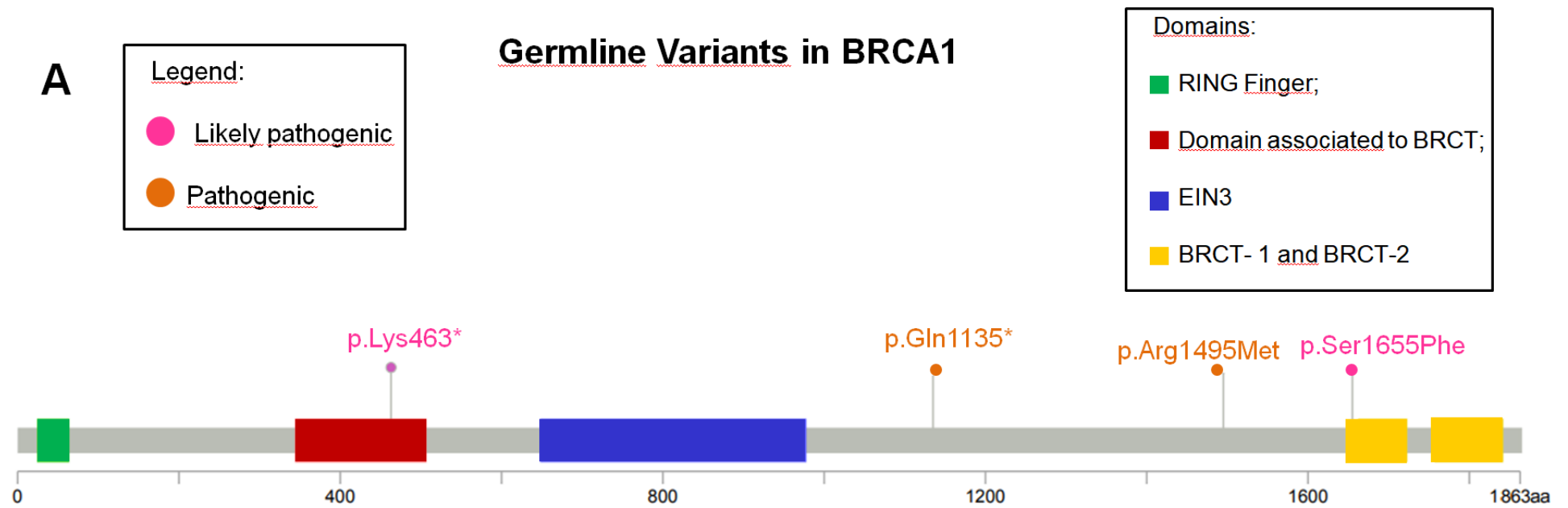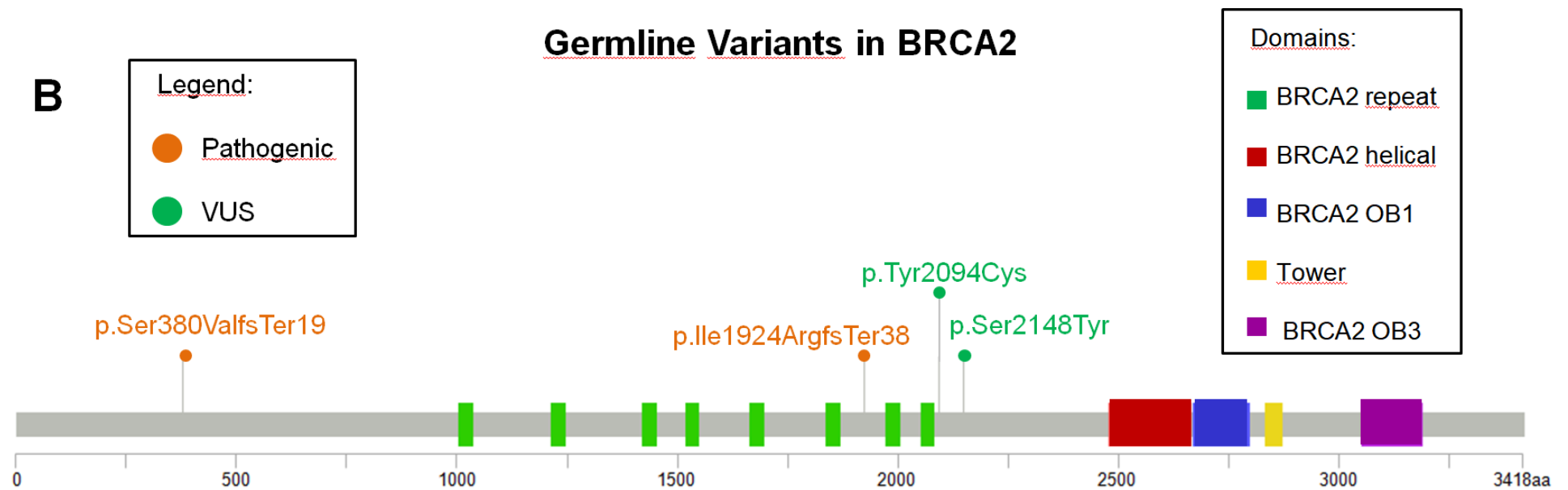

Supplement: Supplementary file 1 — Figure S1 [file CAM4-13-e6729-s001.pdf]

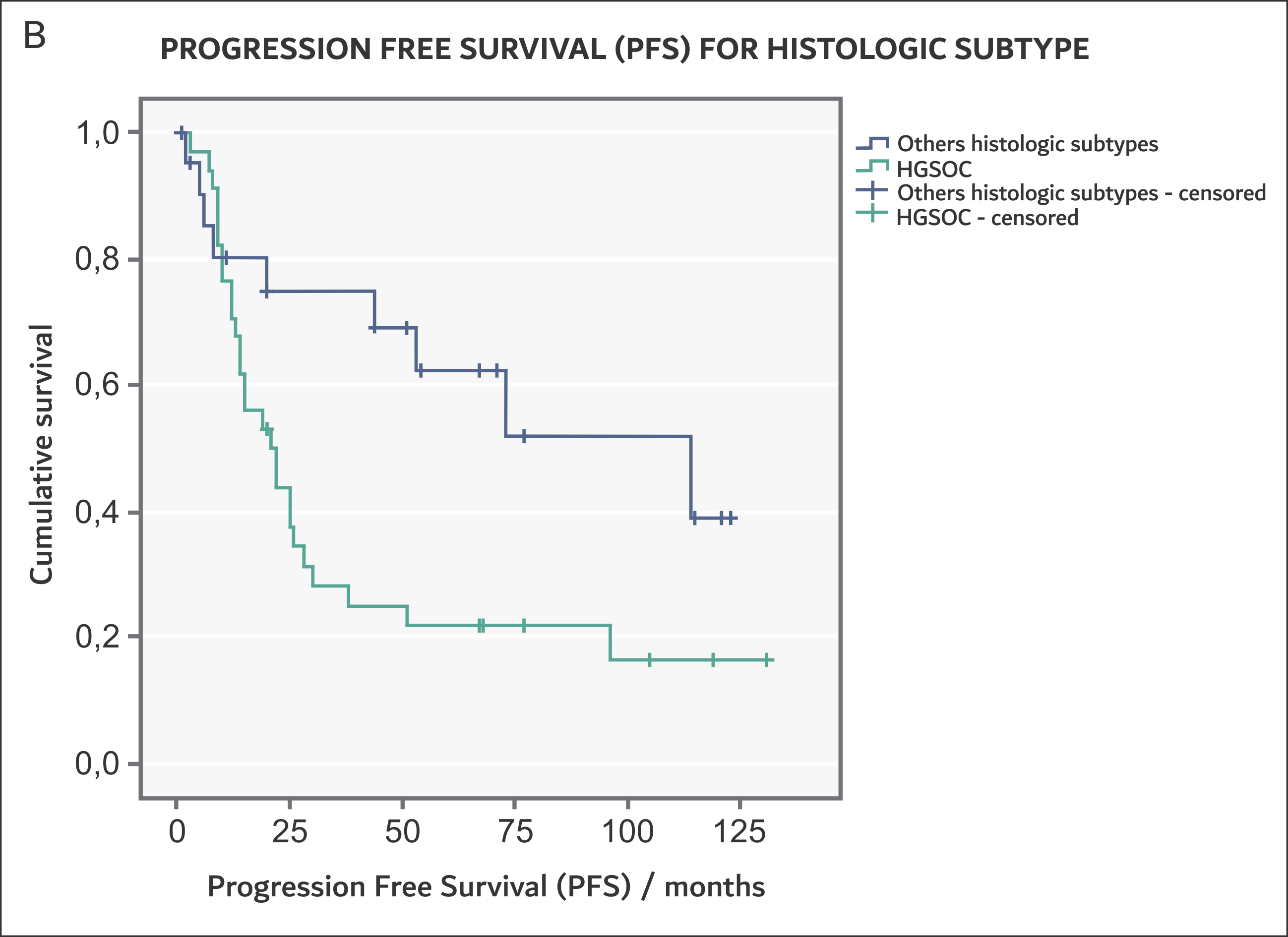

Supplement: Supplementary file 3 — Figure S3 [file CAM4-13-e6729-s005.zip › Supporting Figure 3B revised.tif]

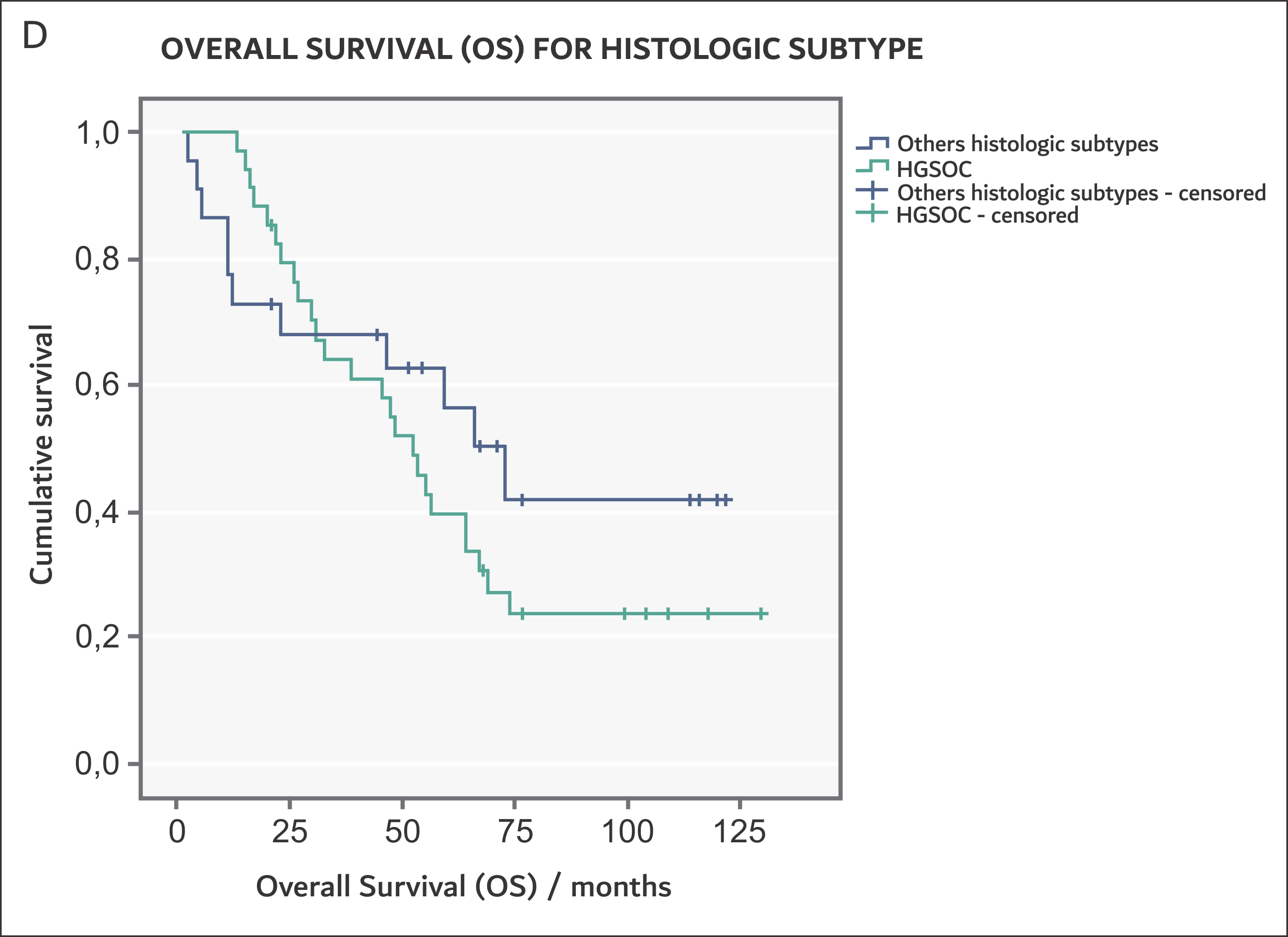

Supplement: Supplementary file 3 — Figure S3 [file CAM4-13-e6729-s005.zip › Supporting Figure 3D revised.tif]

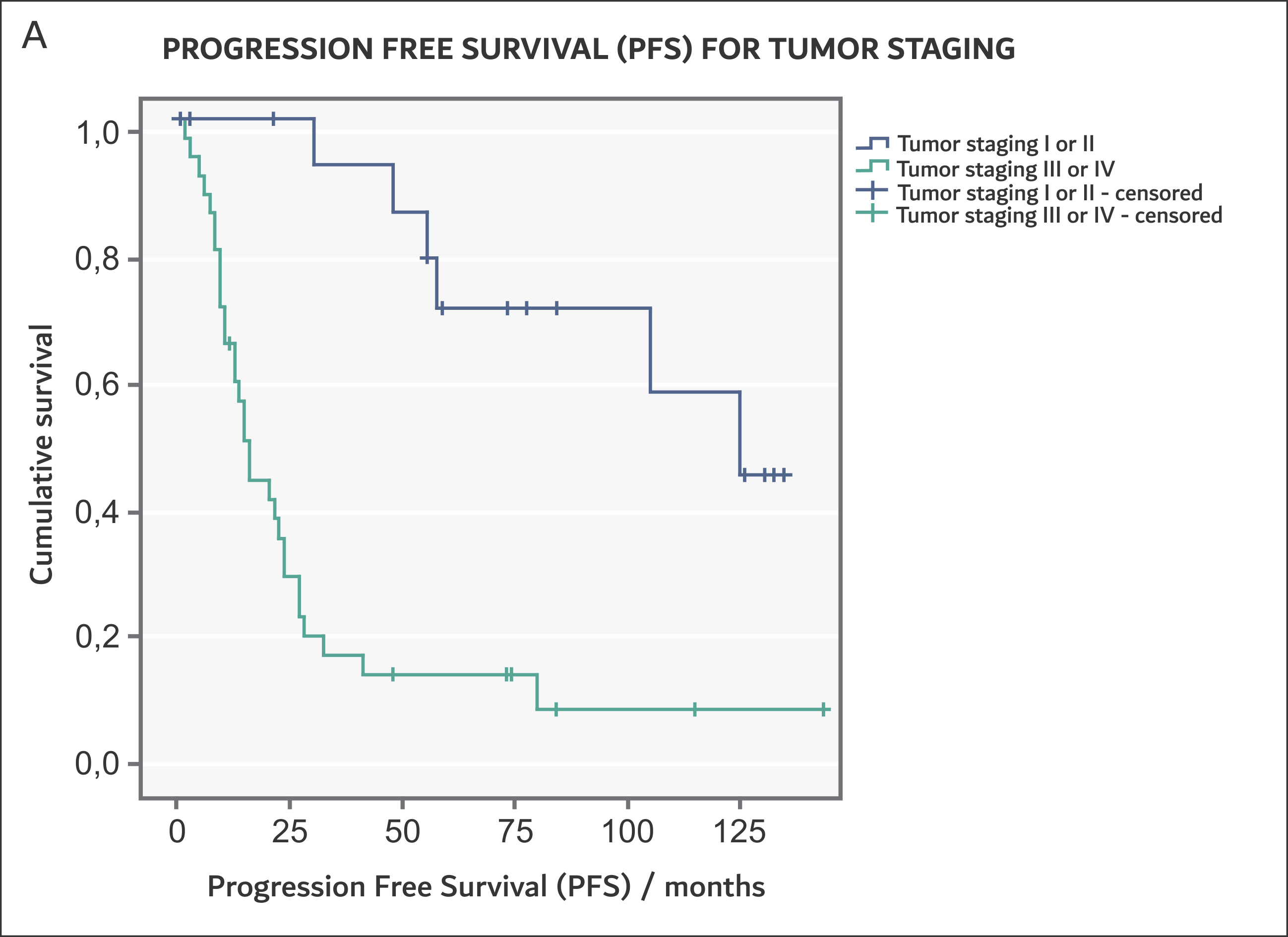

Supplement: Supplementary file 3 — Figure S3 [file CAM4-13-e6729-s005.zip › Supporting Figure 3A revised.tif]

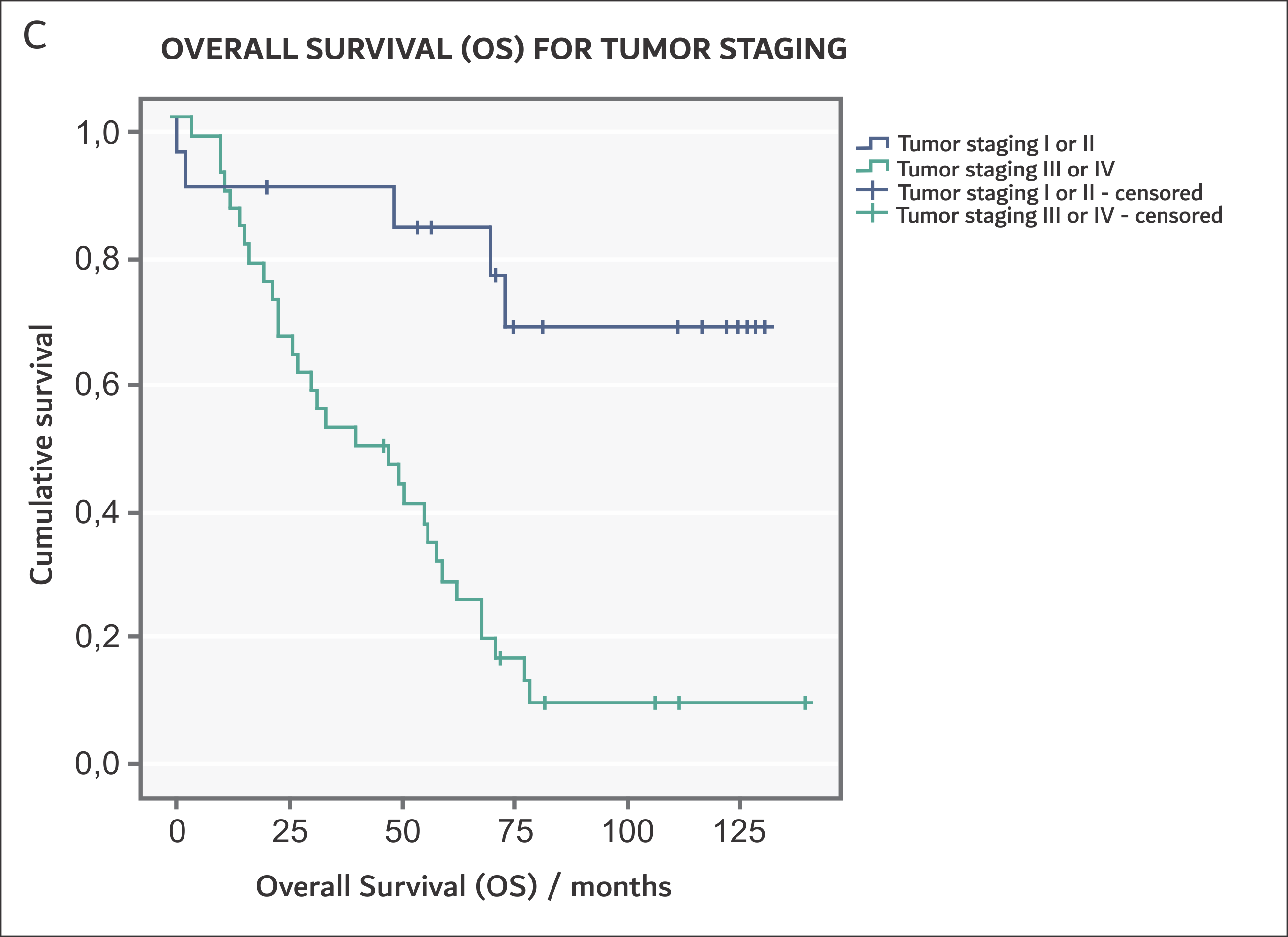

Supplement: Supplementary file 3 — Figure S3 [file CAM4-13-e6729-s005.zip › Supporting Figure 3C revised.tif]
