# Supplementary material for: BRCA1, BRCA2, and TP53 germline and somatic variants and clinicopathological characteristics of Brazilian patients with epithelial ovarian cancer
Source: Cancer Med. 2024 Feb 2;13(3):e6729. doi: 10.1002/cam4.6729 (PMC10905552; doi:10.1002/cam4.6729)
Supplement: Supplementary file 2 — Figure S2 [file CAM4-13-e6729-s003.pdf]

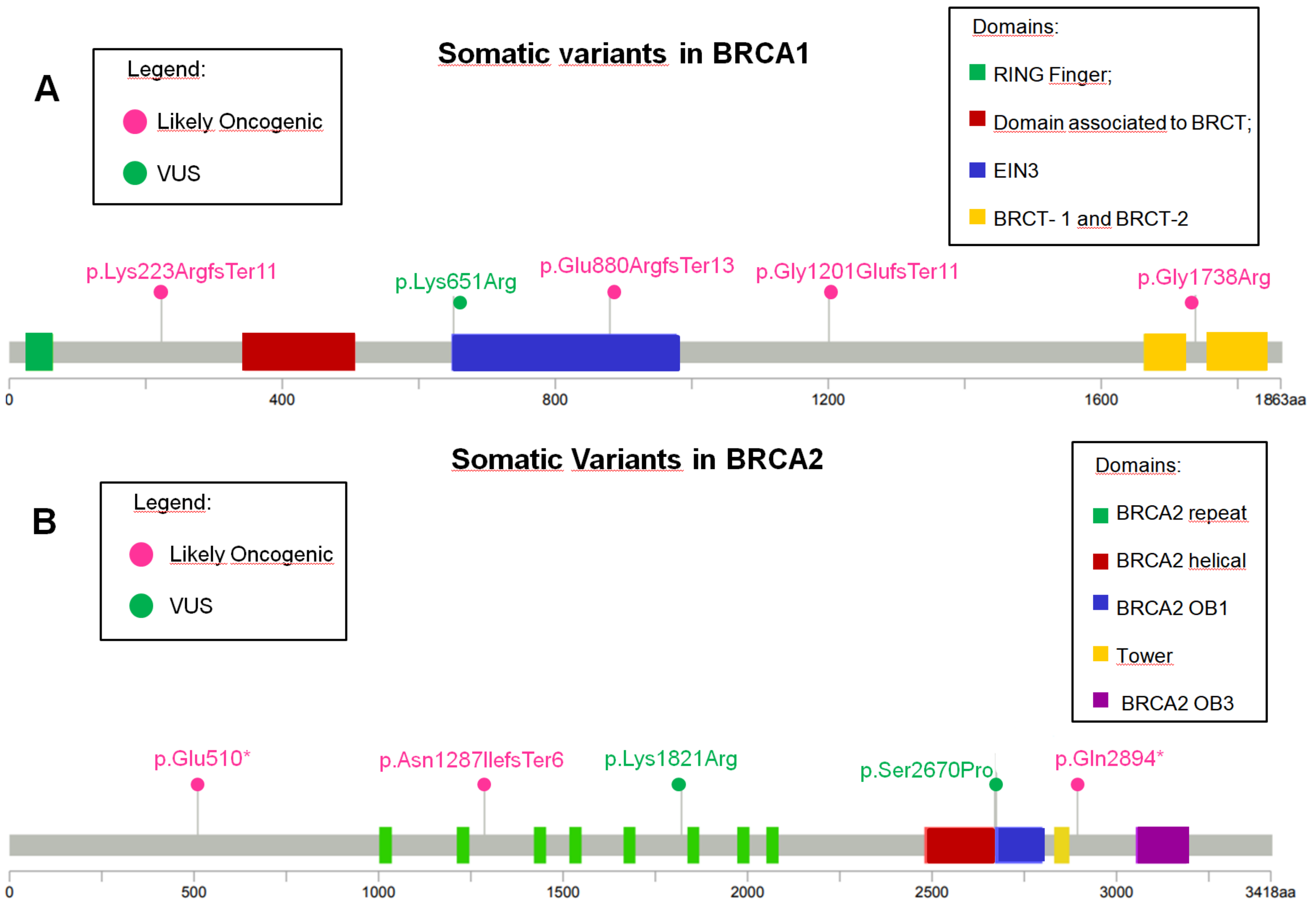
A
Somatic variants in BRCA1
Legend:
Likely Oncogenic
VUS
Domains:
RING Finger;
Domain associated to BRCT;
EIN3
BRCT- 1 and BRCT-2
p.Lys223ArgfsTer11
p.Lys651Arg
p.Glu880ArgfsTer13
p.Gly1201GlufsTer11
p.Gly1738Arg
0
400
800
1200
1600
1863aa
B
Somatic Variants in BRCA2
Legend:
Likely Oncogenic
VUS
Domains:
BRCA2 repeat
BRCA2 helical
BRCA2 OB1
Tower
BRCA2 OB3
p.Glu510*
p.Asn1287IlefsTer6
p.Lys1821Arg
p.Ser2670Pro
p.Gln2894*
0
500
1000
1500
2000
2500
3000
3418aa

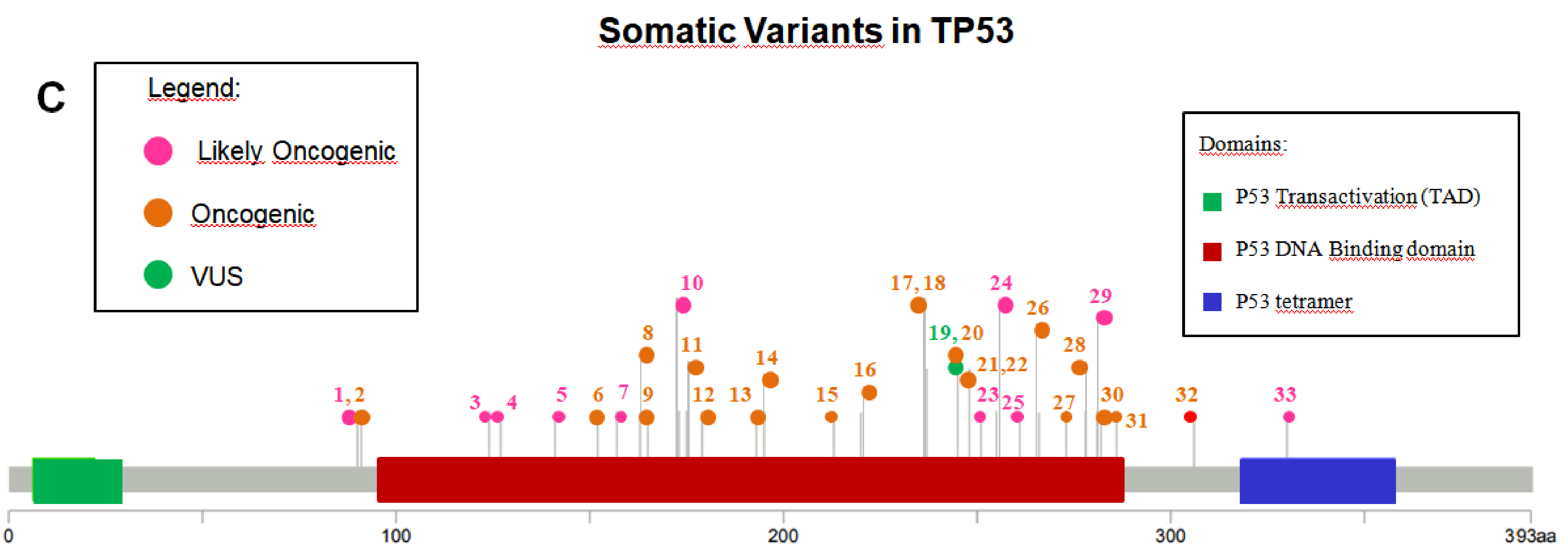

Somatic variants:

1. p.Ser90ProfsTer33;
2. Trp91*;
3. p.Cys124*;
4. p.Ser127Phe;
5. p.Cys141Gly;
6. p.Pro152ArgfsTer18;
7. p.Val157Phe;
8. p.Tyr163Cys;
9. p.Gln165*;
10. p.Val173Leu;
11. p.Arg175His;
12. p.His179Arg;
13. p.His193Arg;
14. p.Ile195Thr;
15. p.Arg213*;
16. p.Tyr220Cys;
17. p.Met237Ile;
18. p.Met237Lys;
19. p.Gly244_Gly245insHisAla;
20. p.Gly245Ser;
21. p.Arg248Gln;
22. p.Arg248Trp;
23. p.Ile251Asn;
24. p.Ile255del;
25. p.Ser261ValfsTer84;
26. p.Gly266Glu;
27. p.Arg273His;
28. p.Pro278Leu;
29. p.Asp281Val;
30. p.Arg282Trp;
31. p.Glu286Lys;
32. p.Arg306*;
33. p.Leu330PhefsTer15
