# Supplementary material for: BRCA1, BRCA2, and TP53 germline and somatic variants and clinicopathological characteristics of Brazilian patients with epithelial ovarian cancer
Source: Cancer Med. 2024 Feb 2;13(3):e6729. doi: 10.1002/cam4.6729 (PMC10905552; doi:10.1002/cam4.6729)
Supplement: Supplementary file 6 — Appendix S3 [file CAM4-13-e6729-s002.docx]

**Supporting Material**

**Appendix S1:** Primers and PCR conditions used to amplify the target region of *BRCA1*, *BRCA2*, and *TP53*.

**Appendix S2:** The germline and somatic variants found in *BRCA1*, *BRCA2*, and *TP53* and characterization of each substitution in respect to pathogenicity and functionality. In addition to information about depth coverage (DC), variant allele frequency (VAF) and tumor representativeness (TR) per sample.

**Figure S1:** Localization of the germline nucleotide variants in respect to protein domains for (A) BRCA1 and (B) BRCA2.

**Figure S2:** Localization of the somatic nucleotide variants in respect to protein domains for (A) BRCA1, (B) BRCA2 and (C) TP53.

**Figure S3:** Kaplan-Meier curves showing the association between (A) tumor staging I-II (n= 19) vs III-IV (n= 37) and (B) histologic subtype others (n= 22) vs HGSOC (n= 34) with progression free survival. Kaplan-Meier curves showing the association between (C) tumor staging I-II (n= 19) vs III-IV (n= 37) and (D) histologic subtype others (n= 22) vs HGSOC (n= 34) with overall survival. Significant associations were found between tumor staging and histologic subtype with progression free survival (p=0.001 and p= 0.018, respectively) and between tumor staging and overall survival (0.001).
